# The Concept of Transmission Coefficient Among Different Cerebellar Layers: A Computational Tool for Analyzing Motor Learning

**DOI:** 10.3389/fncir.2019.00054

**Published:** 2019-08-27

**Authors:** Saeed Solouki, Fariba Bahrami, Mahyar Janahmadi

**Affiliations:** ^1^Control and Intelligent Processing Center of Excellence, Human Motor Control and Computational Neuroscience Laboratory, School of Electrical and Computer Engineering, College of Engineering, University of Tehran, Tehran, Iran; ^2^Department of Physiology, Neuroscience Research Center, School of Medicine, Shahid Beheshti University of Medical Sciences, Tehran, Iran

**Keywords:** transmission coefficient, multi-layer neural network, synaptic plasticity, cerebellar motor learning, optokinetic reflex

## Abstract

High-fidelity regulation of information transmission among cerebellar layers is mainly provided by synaptic plasticity. Therefore, determining the regulatory foundations of synaptic plasticity in the cerebellum and translating them to behavioral output are of great importance. To date, many experimental studies have been carried out in order to clarify the effect of synaptic defects, while targeting a specific signaling pathway in the cerebellar function. However, the contradictory results of these studies at the behavioral level further add to the ambiguity of the problem. Information transmission through firing rate changes in populations of interconnected neurons is one of the most widely accepted principles of neural coding. In this study, while considering the efficacy of synaptic interactions among the cerebellar layers, we propose a firing rate model to realize the concept of transmission coefficient. Thereafter, using a computational approach, we test the effect of different values of transmission coefficient on the gain adaptation of a cerebellar-dependent motor learning task. In conformity with the behavioral data, the proposed model can accurately predict that disruption in different forms of synaptic plasticity does not have the same effect on motor learning. Specifically, impairment in training mechanisms, like in the train-induced LTD in parallel fiber-Purkinje cell synapses, has a significant negative impact on all aspects of learning, including memory formation, transfer, and consolidation, although it does not disrupt basic motor performance. In this regard, the overinduction of parallel fiber-molecular layer interneuron LTP could not prevent motor learning impairment, despite its vital role in preserving the robustness of basic motor performance. In contrast, impairment in plasticity induced by interneurons and background activity of climbing fibers is partly compensable through overinduction of train-induced parallel fiber-Purkinje cell LTD. Additionally, blockade of climbing fiber signaling to the cerebellar cortex, referred to as olivary system lesion, shows the most destructive effect on both motor learning and basic motor performance. Overall, the obtained results from the proposed computational framework are used to provide a map from procedural motor memory formation in the cerebellum. Certainly, the generalization of this concept to other multi-layered networks of the brain requires more physiological and computational researches.

## Introduction

**Figure 1:**
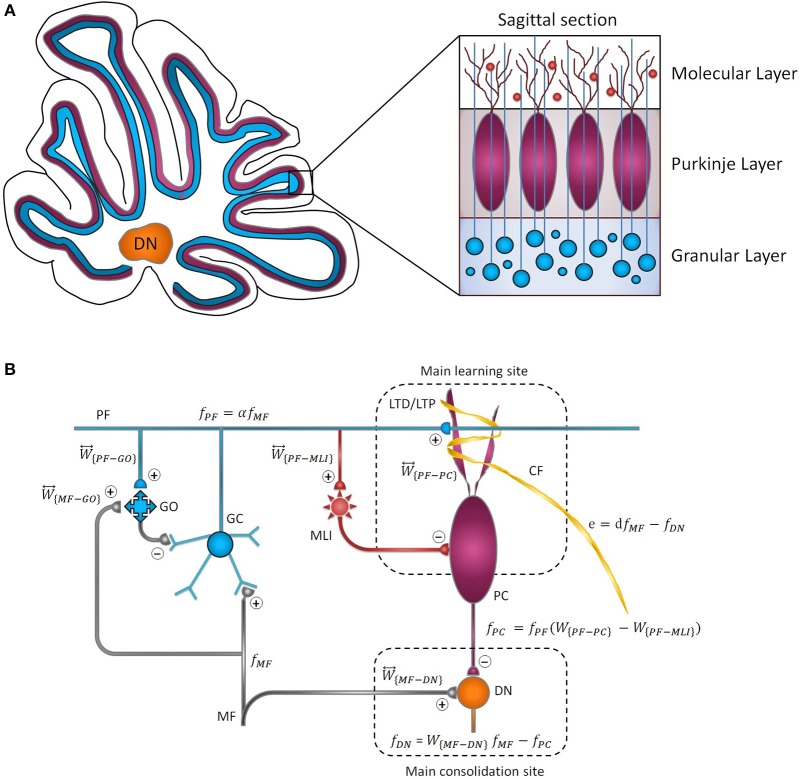
Cytoarchitecture of the cerebellum. **(A)** Cross-sectional views of the cerebellum showing the folding pattern and layered character of the cortex. **(B)** Schematic representation of cerebellar microcircuit. There are two principal afferents to the cerebellar cortex: (1) MFs, which provide sensory information of screen oscillation to GCs, GOs, and DN, and (2) CFs, which convey the retinal image slip information to PCs and drive cortical learning by changing the synaptic efficacy at PF-PC synapses. The ascending axons of the GCs branch in a T-shaped manner to form PFs, which, in turn, make excitatory synaptic contacts with MLIs and PCs. PF-PC and MF-DN synapses are respectively considered as the main sites of learning and consolidation. Synaptic weights are indicated by $\overset{⃡}{W}$.

The cytoarchitecture of the cerebellar cortex has a uniform character and is roughly divided into three parallel circuit elements (Marzban et al., 2015), which, from the inner to the outer layer, are called the granular, the Purkinje, and the molecular layers (Figure 1A). Effective communication between these layers is mainly regulated by synapses (Evans, 2007). Generally, synaptic plasticity has multifaceted roles in the formation, transfer, and consolidation of memory, which are critical for learning, and also for enhancement of signal-to-noise ratio for robust neurotransmission (D'Angelo et al., 2005; Jörntell and Hansel, 2006; Borjkhani et al., 2018). Despite an extensive amount of electrophysiological and behavioral studies on the mechanisms underlying synaptic plasticity (Gao et al., 2012; Freeman, 2015), the precise contribution of each type of plasticity in the evolution of motor memory remains debated (Yuzaki, 2013; D'Angelo et al., 2016), since various parts of the cerebellar circuit are involved in controlling the baseline motor performance, simultaneously integrating multiple cortical and sensory inputs, and modulating the output firing rate of deep nuclei (DN) toward premotor areas (Mauk et al., 2000; Cheron et al., 2013; Badura et al., 2016).

The existence of different forms of synaptic plasticity, besides access to sensorimotor information, has enabled the cerebellum to participate actively in motor learning (De Zeeuw and Ten Brinke, 2015). Motor learning in the cerebellum is mediated by multiple plasticity sites at both cortical and nuclear parts. Naturally, organizing movement patterns in a coordinated manner requires the interaction of different forms of synaptic plasticity in the cerebellar network (Gao et al., 2012). In fact, an individual type of plasticity alone cannot account for wide dynamic ranges of cerebellar learning (Boyden et al., 2004; Solouki and Pooyan, 2016).

As schematically illustrated in Figure 1B, the transmission flow of mossy fibers' (MFs) input to DN consists of two distinct parts: (1) the direct pathway that relays MFs sensory information directly to DN and (2) the indirect or cortical pathway involving MFs, granule cells (GCs), parallel fibers (PFs), and Purkinje cells (PCs). In addition to the mainstream of the cortical pathway, PCs receive inhibitory input from molecular layer interneurons (MLIs). The indirect pathway operates as a fast learning module while deeper structures (direct pathway) work as a slow learning module, wherein the motor skill is transferred and consolidated into more persistent memory (Lee et al., 2015; Luque et al., 2016). The concept of memory formation in the cerebellar cortex and its later transfer to the nuclear tract is supported by the experimental evidence that the flocculus shutdown after four sessions of optokinetic reflex (OKR) adaptation does not impair the previously acquired motor memory (Shutoh et al., 2006). Reversible blockade of floccular protein synthesis by actinomycin or anisomycin microinjection produced a similar effect (Okamoto et al., 2011). The classical theory of cerebellar learning states that repetition of short-term training sessions can gradually lead to persistent changes in the synaptic efficacy (Ito, 2000; Strata, 2009; De Zeeuw and Ten Brinke, 2015). It is postulated that long-term depression (LTD) at PF-PC synapses constitutes the major mechanism of motor learning in the cerebellar cortex (Ito, 2001; Gao et al., 2012; Hirano, 2013). The strength of PF-PC synapses is adjusted under the guidance of climbing fibers (CFs). Thus, the olivary system seems to train the synapses by encoding the error signals that drive cerebellar learning (Carrel, 2012). The presence or absence of CF signal during PF activity causes LTD or LTP, respectively, at the PF-PC synapses. Postsynaptic plasticity at PF to MLI synapses is also mediated, through spillover, by CF activation in the opposite direction of PF-PC plasticity (Hirano, 2013). On the other hand, the plasticity at MF-DN is driven by synchronized activities of MFs and PCs (Gosui and Yamazaki, 2016).

Recently, a controversy arose from the observation in several studies, indicating that although LTD was abrogated genetically or pharmacologically, motor learning in non-anesthetized behaving animals remained unchanged (Welsh et al., 2005; Faulstich et al., 2006; Schonewille et al., 2011; Yuzaki, 2013; Inoshita and Hirano, 2018). To solve the observed controversy, some researchers justified the PF-LTD vs. motor learning mismatch by considering the possibility of a functional or structural compensatory mechanism, which has yet to be proven (Schonewille et al., 2011; Gao et al., 2012). Some others proposed long-term potentiation (LTP) as an alternative substrate for motor learning (Schonewille et al., 2010) and reduced the role of LTD to an inhibitor of synaptic saturation or a protector against excitotoxic PC death during the inferior olive (IO) overactivation (Welsh et al., 2005). One of the reasons of ambiguity in determining the role of LTD and LTP in motor learning is that changes in each one alone, regardless of what happens to the other, are being studied. In most genetic studies, where molecules required for LTD induction were manipulated, LTP alterations have not yet been investigated (Yuzaki, 2013). Furthermore, the deleted gene of interest has a global effect on every cell of the brain and not just the cells of interest. Thus, one cannot be sure that the resulting phenotype is exclusively related to LTD. In the case of discrepancies between PF-LTD and motor learning, more specific experimental or computational analysis are warranted before drawing any final conclusions.

In this study, we take the advantages of organizational uniformity and layered structure of the cerebellum to develop a computational model for investigating the highly distributed nature of cerebellar synaptic plasticity during a motor learning process. Assuming the synapse as a basis for neurotransmission (Stevens, 2003), plasticity is considered as a mechanism for regulating the transmission coefficient among neuronal layers. The main idea behind our computational scheme lies in estimating the effect of altered transmission coefficient among cerebellar layers on the equilibrium behavior of the system. In the first part of the paper, we provide an analytical framework to describe the rate of changes at synaptic weights as a function of population activity in cerebellar neurons and determine five different conjunctions to evaluate the following scenarios:

1. **MF-GC:** The importance of MF-GC synapses in enhancing signal transmission and tuning spike time of GCs on the millisecond order has been shown so far (Garrido et al., 2013b; Sgritta et al., 2017). Here, we decrease the transmission coefficient of the input layer among MFs and GCs to elucidate whether this synapse has any role in motor learning and gain adaptation or not.
2. **PF-PC:** We reevaluate the aforementioned dichotomy between PF-LTD and motor learning by considering the intrinsic differences between the spontaneous LTD, which is induced by background activity of CFs and train-induced LTD, which is triggered by use-dependent activity of CFs. Moreover, we use the model to examine the effect of LTD and LTP blockade on motor learning and baseline motor performance. Meanwhile, we monitor the plastic changes at PF to MLI synapse to determine whether the MLI pathway has any backup role for the PF-PC pathway and, if so, to what extent it would contribute in compensating the LTD/LTP defect at PF-PC synapses. Additionally, our model allowed for a temporally specific ablation of LTD, which helps us to clarify different roles played by the early and late phases of LTD in short- and long-term learning.
3. **IO (CF pathway):** Emerging evidence from experimental studies indicates a high level of coordination between olivary system activity and cerebellar learning (Pijpers et al., 2005; Van Der Giessen et al., 2008; Badura et al., 2013; Solouki et al., 2018). However, it is still unclear which phase of learning will be affected by olivary system disruption. Probably, the extent of functional problems caused by IO lesion would be far beyond the defect of PF-LTD because CFs, which are the axons of IO neurons, are involved in adjusting the synaptic plasticity at both molecular and Purkinje layers. To understand the possible effect of olivary system lesion on cerebellar learning, we simulate learning paradigm in the absence of CF signal.
4. **PF-MLI:** Implication of MLIs in motor learning and the possibility of their synergistic interaction with LTD and LTP at PF-PC synapses are studied from two perspectives. Along with the second scenario, we first examine whether interneurons are able to compensate impairment at PF-PC synapses and, reciprocally, how would impairment at PF-MLI synapses affect PF-PC synaptic plasticity and learning behavior of the model.
5. **PC-DN:** The basis for motor memory consolidation is a controversial topic. One view is that the acquired short-term memory in the cerebellar cortex is transferred to a new location during the learning process, that of so-called saving phenomena (Kellett et al., 2010). In the last scenario, we try to verify the existence of such hierarchical causality by disconnecting the cortical and nuclear parts of the cerebellum.

As a model for cerebellar motor learning, the adaptation of OKR is used. OKR is a kind of cerebellar-dependent oculomotor response that serves to stabilize moving images on the retina (Inoshita and Hirano, 2018). The potential impact of the lesion scenarios on the behavioral trait of the model is addressed by employing the following terms and definitions: (a) basic motor performance, i.e., the initial value of OKR gain before any learning paradigm has taken place; (b) motor learning, which corresponds to adaptation of OKR gain following a learning paradigm; and (c) motor consolidation, which means long-term preservation of the level of adaptation of OKR gain. In the final step, putting together the results of the above scenarios in the execution of a repetitive learning paradigm and tracing their behavioral differences at both phases of short- and long-term learning, we propose a conceptual map of procedural motor memory formation in the cerebellum.

## Materials and Methods

### Definition of Plasticity Rules

Experimental studies in different parts of the brain, including the hippocampus, neocortex, and cerebellum, have revealed activity-dependent processes that can persistently change the efficacies of synapses (Dayan and Abbott, 2001; Gerstner et al., 2014). In this section, we take the advantages of differential equations to characterize the rate of changes of synaptic efficacies as a function of population activity in cerebellar neurons. The transmission delays between the different neural populations are disregarded.

#### Cerebellar Cortex

Looking at Figure 1B, the population firing rate of MF afferents is denoted by $f_{MF}\in R^{n}$. In the context of OKR adaptation, MFs transmit sensory information of visual stimulus to the granular layer and DN. The MF input is then propagated to GCs with an *m* by *n* synaptic connectivity matrix, presumably, *m* ≫ *n*. The GCs are also under the influence of feedforward and feedback inhibition from Golgi cells (GOs). Thus, the output of GCs, denoted by $f_{PF}\in R^{m}$, can be written as follows:

(1) $$\begin{array}{rcl} f_{PF} & = & f_{MF}-W_{\left\{PF-GO\right\}}f_{PF}-W_{\left\{MF-GO\right\}}f_{MF} \end{array}$$

(2) $$\begin{array}{rcl} f_{PF}\left(1+W_{\left\{PF-GO\right\}}\right) & = & f_{MF}\left(1-W_{\left\{MF-GO\right\}}\right) \end{array}$$

(3) $$\begin{array}{rcl} f_{PF} & = & \left(\frac{1-W_{\left\{PF-GO\right\}}}{1+W_{\left\{MF-GO\right\}}}\right)f_{MF}=\alpha f_{MF} \end{array}$$

where *W*_{*MF*−*GO*}_ and *W*_{*PF*−*GO*}_ are constant representing the synaptic weight of MF-GO and PF-GO, respectively. $\alpha =\left(\frac{1-W_{\left\{PF-GO\right\}}}{1+W_{\left\{MF-GO\right\}}}\right)$ is defined as transmission coefficient between excitatory mossy fiber input and granular layer. It is postulated that MF burst activation not only induces presynaptic LTP but also enhances intrinsic excitability of the GCs. This augmented excitability results from an increased input impedance and reduced spike threshold, which heightens excitatory postsynaptic potential (EPSP) and facilitates spike output (Armano et al., 2000; Seja et al., 2012). Thus, despite the low background firing rate of GCs owing to tonic inhibition by GOs, sensory stimulus can evoke bursting in GCs, such that MFs input is transmitted with high reliability to the Purkinje layer (D'Angelo and De Zeeuw, 2009). Moreover, the combination of feedforward and feedback inhibition by GOs opens up a fine-tuned time window for maximum transmission of sensory information with a high signal to noise ratio (Chadderton et al., 2004; Gao et al., 2012). Therefore, the transmission coefficient of the input layer would be ≤ 1 (α ≤ 1). Obviously, this value is higher in the normal group than the lesion one. Herein, α is simply assumed to be near 1 in the normal and 0.3 in the lesion condition.

The excitatory output of GCs is sent to the Purkinje layer by PF axons. Also, the output of each PC is assumed to be *f*_*PC*_ ≡ *W*_{*PF*−*PC*}_*f*_*PF*_ − *W*_{*PF*−*MLI*}_*f*_*PF*_, where $W_{\left\{PF-PC\right\}}\in R\times R^{m}$. The synaptic weight of direct connection among MFs and DN is defined by $W_{\left\{MF-DN\right\}}\in R\times R^{n}$. The final output of the nuclear module (DN) toward the oculomotor neurons is given by *f*_*DN*_ = *W*_{*MF*−*DN*}_*f*_*MF*_ − *f*_*PC*_. The GABAergic nature of PC is taken into account in writing *f*_*DN*_.

The model gradually learns to adapt the actual output *f*_*DN*_ to the desired motor output *df*_*MF*_. If the desired gain *d* is larger (smaller) than the initial state, the synaptic weight experiences depression (potentiation) in *W*_{*PF*−*PC*}_ and potentiation (depression) in *W*_{*MF*−*DN*}_ (Jörntell and Hansel, 2006; Gosui and Yamazaki, 2016). As mentioned in the previous section, CFs pass on the learning error *e* ≡ *df*_*MF*_ − *f*_*DN*_ to PCs, which enables supervised learning at PF-PC synapses. The objective function of supervised learning is given as

(4) $$\begin{array}{r} J\left(W_{\left\{PF-PC\right\}}\right)=\frac{1}{2}e^{2} \end{array}$$

The least mean square (LMS) method can be used to determine the steepest descent direction of the error function (Kawato, 2009):

(5) $$\begin{array}{r} \Delta W_{\left\{PF-PC\right\}}\propto -\frac{\partial J}{\partial W_{\left\{PF-PC\right\}}}=-f_{PF}e=-f_{PF}\left(CF-\bar{CF}\right) \end{array}$$

where $\left(CF-\bar{CF}\right)$ is the difference between the spontaneous and background activity of CFs, assumed to encode the error signal. In the case that both PFs and CFs are activated, *f*_*PF*_ and $\left(CF-\bar{CF}\right)$ are positive, and thus *W*_{*PF*−*PC*}_ is negative (LTD). We can write

(6) $$\begin{array}{r} {\dot{W}}_{\left\{PF-PC\right\}}=-\lambda_{1}\frac{\partial J}{\partial W_{\left\{PF-PC\right\}}}=-\frac{1}{2}\lambda_{1}\frac{\partial e^{2}}{\partial W_{\left\{PF-PC\right\}}}=-\lambda_{1}f_{PF}e \end{array}$$

where λ_1_ is the learning rate. In contrast, lone activation of PFs in the absence of *e*, leads to LTP (Lev-Ram et al., 2002; Coesmans et al., 2004; Hirano, 2013). We model this effect by adding λ_2_*f*_*PF*_ to Equation (6) (Dayan and Abbott, 2001). Although this subtractive normalization term is used to contract the train-induced LTD, it cannot avoid the saturation of *W*_{*PF*−*PC*}_ when the error signal is off. In the absence of error signal, the spontaneous activity of PFs is counterbalanced by background activity of CF (Ito, 2013). Thus, we decrease the magnitude of *W*_{*PF*−*PC*}_ by considering the effect of spontaneous LTD, which is denoted by −λ_2_*LTD*_*spon*_. This subtle definition helps us to examine the defects in train-induced and spontaneous LTD, separately. We also add λ_3_*W*_{*PF*−*PC*}_ as a synaptic decaying factor (Dayan and Abbott, 2001; Gerstner et al., 2014). Eventually, Equation (6) is turned to

(7) $$\begin{array}{l} {\dot{W}}_{\left\{PF-PC\right\}}=-\overset{train-induced\;LTD}{\overset{︷}{\lambda_{1}\alpha f_{MF}(df_{MF}-W_{\left\{MF-DN\right\}}f_{MF}-W_{\left\{PF-MLI\right\}}}}\\ \;\alpha f_{MF}+W_{\left\{PF-PC\right\}}\alpha f_{MF})\\ \;+\overset{spontaneus\;LTP}{\overset{︷}{\lambda_{2}\alpha f_{MF}}}-\overset{spontaneus\;LTD}{\overset{︷}{\lambda_{2}LTD_{spon}}}-\lambda_{3}W_{\left\{PF-PC\right\}} \end{array}$$

where λ_2_ and λ_3_ are the recovery rates satisfying λ_1_ ≫ λ_2_, λ_3_. In the rest or post-training period, the OKR gain, which might have amplified with adaptation, tends back to its initial value. Let us indicate this initial gain by *d* = *d*_0_. In this null condition, the synaptic weights return to their baseline $\left(W_{\left\{PF-PC\right\}}^{0},W_{\left\{PF-MLI\right\}}^{0},\;W_{\left\{MF-DN\right\}}^{0}\right)$ and the error signal approaches zero. Thus, by setting Ẇ_{*PF*−*PC*}_ = 0 in the above equation, we derive

(8) $$\begin{array}{l} \lambda_{2}(\alpha f_{MF}-LTD_{spon})=\lambda_{1}\alpha f_{MF}\overset{e\to 0}{\overset{︷}{\begin{array}{l} (d_{0}f_{MF}-W_{\left\{MF-DN\right\}}^{0}f_{MF}-W_{\left\{PF-MLI\right\}}^{0}\\ \;\alpha f_{MF}+W_{\left\{PF-PC\right\}}^{0}\alpha f_{MF}) \end{array}}}\\ \;+\;\lambda_{3}W_{\left\{PF-PC\right\}}^{0} \end{array}$$

As seen, the balance between PF-LTD and PF-LTP induced by the spontaneous activity of CF and PFs is equal to the baseline weight of *W*_{*PF* − *PC*}_. Replacing λ_2_(α*f*_*MF*_−*LTD*_*spon*_) with $\lambda_{3}W_{\left\{PF-PC\right\}}^{0}$ in Equation (7) and considering a regulatory coefficient (β_1_) for manipulating the strength of train-induced LTD results in

(9) $$\begin{array}{l} {\dot{W}}_{\left\{PF-PC\right\}}=-\lambda_{1}\beta_{1}\alpha f_{MF}(df_{MF}-\;W_{\left\{MF-DN\right\}}f_{MF}\\ \;-W_{\left\{PF-MLI\right\}}\alpha f_{MF}+W_{\left\{PF-PC\right\}}\alpha f_{MF})\\ \;-\;\lambda_{3}(W_{\left\{PF-PC\right\}}-W_{\left\{PF-PC\right\}}^{0}) \end{array}$$

The other neurons stimulated by PFs are MLIs. As mentioned in the previous section, PF-MLI synapses are suggested to exhibit bidirectional plasticity in the opposite direction to that induced at PF-PC synapses. Coherent activation of PFs and a CF induces LTP, whereas lone stimulation of PFs induces LTD at PF-MLI synapses. Thus, changing the direction of LTP and LTD in Equation (9) yields the following plasticity rule for PF-MLI synapse.

(10) $$\begin{array}{l} {\dot{W}}_{\left\{PF-MLI\right\}}=+\;\lambda_{1}\beta_{3}\alpha f_{MF}(df_{MF}-W_{\left\{MF-DN\right\}}f_{MF}\\ \;\;-W_{\left\{PF-MLI\right\}}\alpha f_{MF}+W_{\left\{PF-PC\right\}}\alpha f_{MF})\\ \;{-\lambda }_{3}(W_{\left\{PF-MLI\right\}}-W_{\left\{PF-MLI\right\}}^{0}) \end{array}$$

where β_3_ is a regulatory coefficient for manipulating the strength of train-induced MLI-LTP and $W_{\left\{PF-MLI\right\}}^{0}$ is the baseline weight of PF-MLI synapse. Given the indirect relation of MLIs and CFs through spillover, the amount of use-dependent synaptic weight changes at PF-MLI synapses is not as large as that at PF-PC synapses. Thus, the default value of β_3_ is smaller than β_1_. It should be noted that in the third scenario, when we are examining IO lesion, β_1_ *and β*_3_ are replaced by β_2_.

#### Cerebellar Nuclei

The activity of DN has critical impact on precise modulation of long-term learning. However, there have been few biological studies that explain the nuclear plasticity changes in the face of upstream signals (D'Angelo et al., 2016). Numerical models predict that the potentiation in DN could be driven by PC output (Pugh and Raman, 2006; Garrido et al., 2013a; Solouki et al., 2019). We also know that MFs are the only excitatory inputs that induce LTP in nucleus synapses. Indeed, large activity of MFs together with the small activity of PCs should reasonably induce LTP at MF-DN synapses (Pugh and Raman, 2006). Taking into account the above facts, we model LTP in an associative manner as a general function of pre- and post-synaptic activities in DN (*H*(*f*_*MF*_, *f*_*DN*_, *f*_*PC*_)). It is also observed that the sole activation of MFs induces LTD in *W*_{*MF*−*DN*}_ (Pugh and Raman, 2006; Hirano, 2013). A subtractive normalization term (λ_5_*f*_*MF*_) is used to show this effect (Gerstner et al., 2014). The magnitude of *W*_{*MF*−*DN*}_ is also constrained by adding a multiplicative decaying factor (λ_6_*W*_{*MF*−*DN*}_) (Gerstner et al., 2014). Finally, Ẇ_{*MF*−*DN*}_ can be written as

(11) $$\begin{array}{r} {\dot{W}}_{\left\{MF-DN\right\}}=\lambda_{4}H\left(f_{MF},f_{DN},f_{PC}\right)-\lambda_{5}f_{MF}-\lambda_{6}W_{\left\{MF-DN\right\}} \end{array}$$

Presumably, *W*_{*MF*−*DN*}_ represents the slow dynamics and evolves much more slowly than *W*_{*PF*−*PC*}_. Therefore, we assume λ_4_, λ_5_, λ_6_ ≪ λ_1_.

Numerical simulations have revealed that the synaptic weight of MF-DN is liable to induce a kind of Hebbian plasticity with postsynaptic gaiting mechanism (Medina and Mauk, 2000; Kawato, 2009; Mapelli et al., 2015). This gaiting is mainly regulated by two afferents to DN, namely, *f*_*PC*_ and *f*_*MF*_. Logically, LTP occurs when *f*_*MF*_ is large and *f*_*PC*_ is small. This can be denoted in the following way (Gerstner et al., 2014):

(12) $$\begin{array}{r} \Delta W_{\left\{MF-DN\right\}}\propto f_{MF}\left(DN-\bar{DN}\right)\;or\;f_{MF}\left(f_{PC}^{\max}-f_{PC}\right) \end{array}$$

where $\bar{DN}$ is the background firing rate (temporal average) of DN and $f_{PC}^{\max}$ is the maximum firing rate of PC. Let us set $H=\;f_{MF}\left(\;f_{PC}^{\max}-f_{PC}\right)$, then, Equation (11) becomes

(13) $$\begin{array}{l} {\dot{W}}_{\left\{MF-DN\right\}}=\lambda_{4}f_{MF}(f_{PC}^{\max}-W_{\left\{PF-PC\right\}}\alpha f_{MF}\\ \;+\;W_{\left\{PF-MLI\right\}}\alpha f_{MF})-\lambda_{5}f_{MF}-\lambda_{6}W_{\left\{MF-DN\right\}} \end{array}$$

With the null condition denoted by *Ẇ*_{*MF*−*DN*}_ = 0 and $\left(W_{\left\{PF-PC\right\}}^{0},W_{\left\{PF-MLI\right\}}^{0},\;W_{\left\{MF-DN\right\}}^{0}\right)$, the subtractive normalization can be specified as follows:

(14) $$\begin{array}{l} \lambda_{5}f_{MF}=\lambda_{4}f_{MF}(f_{PC}^{\max}-W_{\left\{PF-PC\right\}}^{0}\alpha f_{MF}+W_{\left\{PF-MLI\right\}}^{0}\alpha f_{MF})\\ \;-\;\lambda_{6}W_{\left\{MF-DN\right\}}^{0} \end{array}$$

Substituting Equation (14) into Equation (13), besides inserting a regulatory coefficient (γ) for manipulating the strength of corticonuclear tract connectivity in the first term of Equation (13), yields.

(15) $$\begin{array}{l} {\dot{W}}_{\left\{MF-DN\right\}}=\lambda_{4}\gamma (W_{\left\{PF-MLI\right\}}-W_{\left\{PF-PC\right\}}+W_{\left\{PF-PC\right\}}^{0}\\ \;-W_{\left\{PF-MLI\right\}}^{0})\alpha f_{MF}{}^{2}\\ \;+\;\lambda_{6}(W_{\left\{MF-DN\right\}}^{0}-W_{\left\{MF-DN\right\}}) \end{array}$$

## Learning Protocol and Simulation Setup

**Figure 2:**
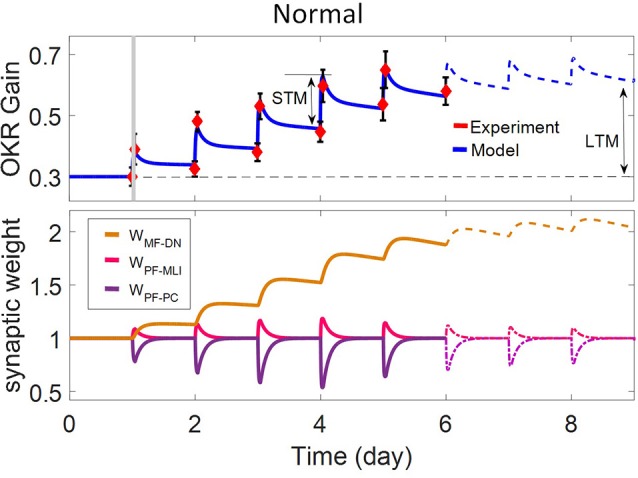
Simulation of OKR adaptation in normal condition. OKR gain changes **(top)** and evolution of synaptic weights at PF-PC, PF-MLI, and MF-DN **(bottom)** during eight sessions of training. The dashed black line indicates the initial level of OKR gain before any training has taken place. The amounts of short- and long-term learning for the 4th and 8th days are indicated by STM and LTM labels. The shaded region specifies the 1-h training period on the first day. The learning curve of the model for the last 3 days, in the absence of experimental data, is plotted by a dashed line.

In this study, we utilized a typical learning protocol proposed by Shutoh et al. (2006). They recorded horizontal OKR gain of fixed head mice in response to sustained oscillation of checked-pattern screen. The behavioral data together with the simulation results for the normal case are depicted in Figure 2. Learning simulation is conducted in a course of five sessions; each contains 1 h of training and 23 h of post-training off-task period. Long-term (slow) adaptation is estimated by gain differences before training on the first day and after training on each day. Likewise, short-term (fast) adaptation is measured by gain differences before and after 1-h daily training. Other experimental data from Katoh et al. (1998) and Shutoh et al. (2006) are used to verify the results of the model in a lesion case. The former investigated the role of chronic olivary system lesions on short- and long-term adaptation of OKR and the latter investigated the effect of reversible flocculus lesion on the transsynaptic shift in memory trace from cerebellar cortex to DN by injecting lidocaine into the cerebellar cortex of mice immediately after training on the 4th day.

Simulation of lesion scenarios in the current study is carried out by manipulating the embedded coefficients in the updating rules (α, β, and γ) and modifying the corresponding baseline values of synaptic weighs ($W_{\left\{PF-PC\right\}}^{0}$, $W_{\left\{PF-MLI\right\}}^{0}$, and $W_{\left\{MF-DN\right\}}^{0}$). In the first case, we partially attenuate MF-GC transmission by reducing α from 1 to 0.3. In the second case, we implement a multi-stage plan to investigate the role of a) train-induced LTD, b) spontaneous LTD, c) spontaneous LTP, and d) learning and recovery rate at PF-PC synapse, separately. Partial and complete blockade of train-induced PF-PC LTD is done by reducing β_1_ from 1 to 0.3 and 0, respectively. According to Equation (7), spontaneous PF-PC LTD deficit disturbs the baseline balance of *W*_{*PF*−*PC*}_ in favor of spontaneous LTP and increases *W*_{*PF*−*PC*}_. From Equations (12) and (15), the growth of *W*_{*PF*−*PC*}_ and subsequent increased activity of PCs takes *W*_{*MF*−*DN*}_(*t*) to the resting state. Moreover, from Equation (10), the increase of *W*_{*PF*−*PC*}_ and the decrease of *W*_{*MF*−*DN*}_ enlarge *W*_{*PF*−*MLI*}_. Therefore, to simulate the impairment of spontaneous PF-PC LTD, we set $W_{\left\{PF-PC\right\}}^{0}=W_{\left\{PF-MLI\right\}}^{0}=1.1$ and $W_{\left\{MF-DN\right\}}^{0}=0$, which were set at 1 in the normal condition. In contrast to spontaneous LTD, impairment of spontaneous PF-PC LTP decreases the synaptic weight of *W*_{*PF*−*PC*}_ toward LTD until it stops at 0 (Equation 7). Under this condition, the baseline values of *W*_{*MF*−*DN*}_ and *W*_{*PF*−*MLI*}_ shift to a lower level (Equations 10 and 15). In practice, vanishing all PF-PC synapses reduces the working frequency and basal intrinsic excitability of PCs, which in turn affects the activity of MLIs and DN (Schonewille et al., 2010). To simulate the impairment of spontaneous PF-PC LTP, we set $W_{\left\{PF-PC\right\}}^{0}=\beta_{1}=\lambda_{3}=0$, β_3_ = 0.48, and $W_{\left\{PF-MLI\right\}}^{0}=W_{\left\{MF-DN\right\}}^{0}=0.5$. Finally, we carry out simulation with a different set of learning and recovery rate to determine the impact of early and late phases of LTD induction on short- and long-term learning and to check the robustness of suggested plasticity rule against parameter variations. In the third case, we blockade CF signaling to cerebellar cortex by replacing β_1_ and β_3_ with β_2_. Partial and complete blockade of CF signal is simulated by setting β_2_ at 0.3 and 0, respectively. In the fourth case, we block either plasticity or baseline activity of PF-MLI synapses. In the case of no PF-MLI plasticity, *W*_{*PF*−*MLI*}_ remains constant at its baseline level, that is, $W_{\left\{PF-MLI\right\}}\left(t\right)=W_{\left\{PF-MLI\right\}}^{0}$ for any *t*. Elimination of PF-MLI baseline activity is also made by setting $W_{\left\{PF-MLI\right\}}^{0}=0$. This manipulation reinforces the inhibition from PCs to DN and subsequently reduces *W*_{*MF*−*DN*}_(*t*) so that $W_{\left\{MF-DN\right\}}^{0}=0$. The overactivation of PCs in the absence of PF-MLI baseline is compensated by decreasing the baseline of *W*_{*PF*−*PC*}_ from 1 to 0.4 and setting β_1_ = 0.1. Furthermore, to avoid LTP saturation at PF-MLI synapses, β_3_ is set at 0.2. In the last scenario, we block PC-DN transmission by setting γ = 0 immediately after training on the fourth session of learning.

We adjust the time constants (τ) of induction and recovery periods, which are equal to the inverse of learning rates (λ), based on the electrophysiological study done by Le et al. (2010). They demonstrated that the induction of LTD or LTP at PF-PC synapses occurs within 5–30 min and the recovery from training takes more than 1 day. It is also reported that OKR adaptation is capable to induce a 30–40% decrease in the amplitude of PF excitatory postsynaptic current (PF-EPSC) or the rising slope of PF-EPSP in PCs (Schonewille et al., 2011; Inoshita and Hirano, 2018). Based on the above evidence, a relatively specific and limited space was searched to find the most appropriate set of learning rates for fitting simulation results with the experimental data. Specifically, the values of λ_2_ and λ_5_ are simply determined by substituting the initial conditions into Equations (7) and (13), respectively. In addition, the baseline values of *W*_{*PF*−*PC*}_ and *W*_{*MF*−*DN*}_ ($W_{\left\{PF-PC\right\}}^{0},W_{\left\{MF-DN\right\}}^{0}$) are achieved by the counterbalance between PF-LTD and PF-LTP from Equations (8) and (14).

Based on the fact that the final output of the cerebellum to the oculomotor neurons is provided by DN (Figure 1B), a portion of its modular activity, mediated by the upstream synaptic weights *W*_{*PF*−*PC*}_, *W*_{*PF*−*MLI*}_, and *W*_{*MF*−*DN*}_, is associated with the OKR adaptation (Mapelli et al., 2015; Gosui and Yamazaki, 2016). Therefore, a normalization factor is used to transform the actual gain from cerebellar space (*W*_{*MF*−*DN*}_ − *W*_{*PF*−*PC*}_α + *W*_{*PF*−*MLI*}_α) to the real world.

(16) $$\begin{array}{r} G_{OKR}=g_{OKR0}\left(W_{\left\{MF-DN\right\}}-W_{\left\{PF-PC\right\}}\alpha +W_{\left\{PF-MLI\right\}}\alpha \right) \end{array}$$

where g_OKR0_ is a scaling factor used to adjust the initial amplitude of OKR. Regarding the initial state of the behavioral data of normal case (Figure 2), we set *g*_*OKR*0_ = 0.3. Further details about the model parameters of the simulated scenarios can be found in Table 1.

**Table 1:** Summary of model parameters for the simulated scenarios.

**Model parameters**	**Simulation scenario**									
	**Normal**	**Attenuation of MF-GC transmission**	**Blockade of train-induced** **PF-PC LTD**	**Blockade of spontaneous** **PF-PC LTD**	**Blockade of spontaneous** **PF-PC LTP**	**Alteration the rate of LTD induction at PF-PC synapse**	**Blockade of CF signal to cerebellar cortex**	**Blockade of PF-MLI plasticity**	**Blockade of PF-MLI baseline activity**	**Blockade of PC-DN transmission**
α	1	0.3	1	1	1	1	1	1	1	1
β_1_	1	1	0.3, 0^a^	1	0	1	–	1	0.1	1
β_2_	–	–	–	–	–	–	0.3, 0^a^	–	–	–
β_3_	0.5	0.5	0.5	0.5	0.48	0.5	–	0	0.2	0.5
γ	1	1	1	1	1	1	1	1	1	0^b^
$W_{\left\{PF-PC\right\}}^{0}$	1	1	1	1.1	0	1	1	1	0.4	1
$W_{\left\{PF-MLI\right\}}^{0}$	1	1	1	1.1	0.5	1	1	1	0	1
$W_{\left\{MF-Dn\right\}}^{0}$	1	1	1	0	0.5	1	1	1	0	1
$\tau_{1}^{\text{c}}$	0.33	0.33	0.33	0.33	0.33	2	0.33	0.33	0.33	0.33
$\tau_{3}^{\text{c}}$	2.5	2.5	2.5	2.5	≫2.5^d^	1.5	2.5	2.5	2.5	2.5
$\tau_{4}^{\text{c}}$	5.5	5.5	5.5	5.5	5.5	5.5	5.5	5.5	5.5	5.5

a *The coefficients of 0.3 and 0 are applied in the cases of partial and complete blockade, respectively*.

b *The value of γ is set at 0 immediately after training on the fourth session*.

c *Time constant (τ_i_) is equal to the inverse of learning rate (λ_i_) and expressed in hours. τ_1_ and τ_3_ represent time constant of W_{PF−PC}_ during learning and recovery period, respectively. The values of τ_4_ and τ_6_, which represent the time constant of W_{MF−DN}_ during and after training, are considered equal*.

d *The value of τ_3_ can be arbitrarily so large that λ_3_ = 0*.

## Results

**Figure 3:**
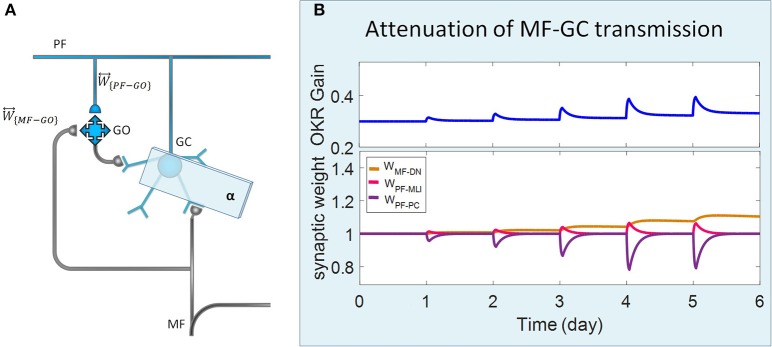
Lesion in the transmission of the granular layer. **(A)** Schematic representation of lesion location in the granular layer. **(B)** Simulated OKR adaptation **(top)** and evolution of synaptic weights **(bottom)** during five sessions of training, while the transmission coefficient of MF-GC synapses (α) is attenuated from 1 to 0.3.

**Figure 4:**
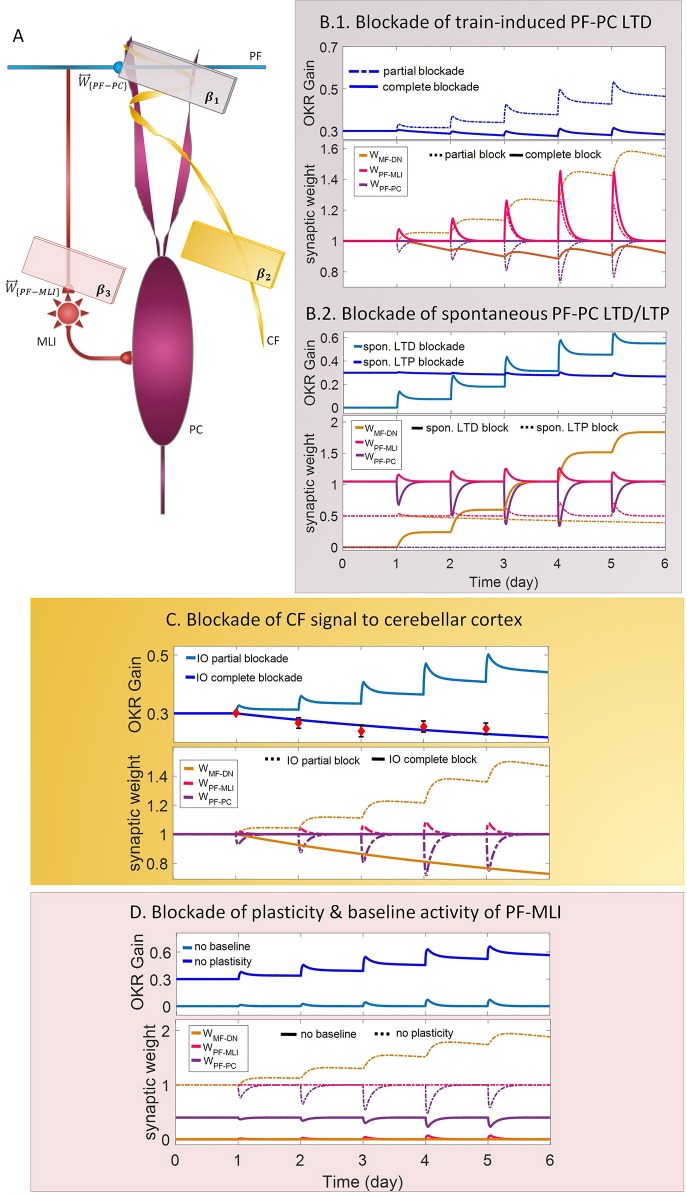
Lesion in the transmission of the molecular and the Purkinje layers. **(A)** Schematic representation of lesion sites and the corresponding transmission coefficients. **(B–D)** Dynamics of the model in gain-up training for 5 days. Each panel consists of the learning curve(s) of OKR gain at the top and its corresponding time-dependent changes in synaptic weights at the bottom. Experimental data in **(C)** are plotted in red dots.

**Figure 5:**
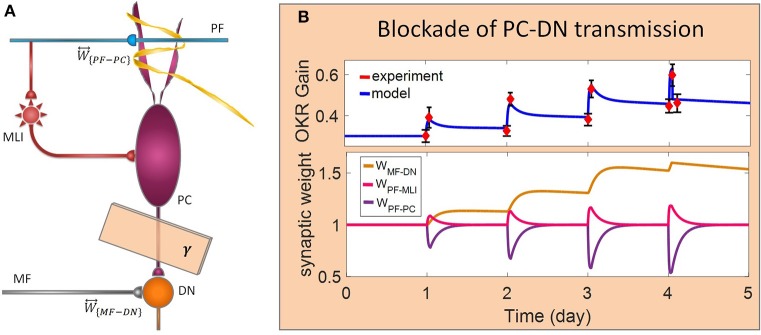
Lesion in the transsynaptic shift of memory trace from cerebellar cortex to DN. **(A)** Schematic representation of lesion location. **(B)** Dynamics of the model in gain-up training for 4 days. Lesion is made by interruption of PC-DN transmission immediately after 1-h training on the 4th day. Experimental data are plotted in red dots.

The dynamics of the present model related to the normal and lesion scenarios are plotted in six panels (Figures 2–**5**). Each panel consists of the learning curve(s) of OKR gain at the top and its corresponding time-dependent changes in weights of PF-PC (*W*_{*PF*−*PC*}_), PF-MLI (*W*_{*PF*−*MLI*}_), and MF-DN (*W*_{*MF*−*DN*}_) synapses at the bottom view. Initially, we assess the model capability to reproduce the observed OKR gain adaptation in wild-type mouse (Figure 2). It can be seen that the daily repetition of training sessions increased the overall gain step by step from 0.3 to 0.56 after 5 days. When the simulation proceeded for 3 more days, OKR gain further increased at a slower pace until it reached an equilibrium point. The amplitude of OKR increased rapidly during 1 h of sustained daily training and diminished throughout post training or rest period, but did not come back to its previous level. Obviously, the rapid changes are related to the fast dynamic (*W*_{*PF*−*PC*}_) and the gradual low changes at the end of each day relative to the start of the same day are attributed to slow dynamic (*W*_{*MF*−*DN*}_). The results also support the idea of distributed and synergistic interaction of different forms of plasticity in the cerebellum during a motor learning task (Boyden et al., 2004; Gao et al., 2012; Garrido et al., 2013a; Solouki and Pooyan, 2016). It can be seen that adaptation to a larger gain effectively increases the value of *W*_{*MF*−*DN*}_, while it does not change the final value of *W*_{*PF*−*PC*}_. Facing the error signal during the training condition (*d* > *d*_0_), *W*_{*PF*−*PC*}_ undergoes LTD and then recovers to its initial value in the dark condition. It seems that what has been learned by *W*_{*PF*−*PC*}_ during training is partially transferred to *W*_{*MF*−*DN*}_. Therefore, *W*_{*PF*−*PC*}_ and *W*_{*MF*−*DN*}_ are suggested to be responsible for short-term memory formation (STM) and long-term consolidation (LTM), respectively.

*W*_{*PF*−*MLI*}_ also changes in sync with *W*_{*PF*−*PC*}_ but in the opposite direction. The GABAergic nature of interneurons converses the PF-MLI plasticity to the same direction as the PF-PC plasticity. This increases the possibility of *W*_{*PF*−*MLI*}_ participation in STM and compensation of *W*_{*PF*−*PC*}_, which is investigated in the next scenarios. Additionally, the depth of modulation in *W*_{*PF*−*PC*}_ reaches its maximum level after 3 days. Theoretically, this seems unusual since |e| is the highest on the first day. In practice, however, the long-term gain of OKR increases in an S shape (Shutoh et al., 2006; Takeuchi et al., 2008; Inoshita and Hirano, 2018). We show this effect by adding a regulatory factor to the first term of Equation (9) just for the first 3 days. To simulate OKR adaptation in the lesion conditions, we modified simulation settings including the transmission coefficients highlighted in Figures 3–5 and baseline values of synaptic weights for compensation, which may occur in behaving animals (see details in Table 1).

**Figure 6:**
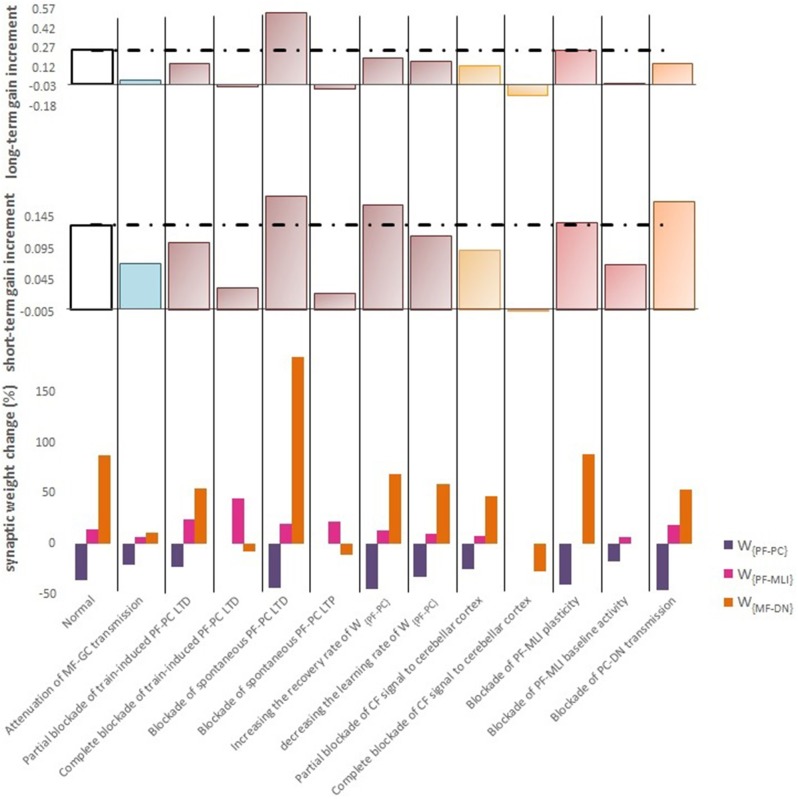
Quantitative data of the model's behavior in confronting different lesion scenarios. **(Top)** Bar graph displaying the long-term or slow adaptation of OKR gain measured by gain differences after training on the 5th day and before training on the first day. Positive and negative long-term gain increments are respectively, considered as the occurrence of motor learning and disruption of basic motor performance. Blockade of CF signaling to cerebellar cortex had the most destructive effect on both motor learning and basic motor performance. Conversely, blockade of PF-MLI plasticity had the least negative impact on the increment of OKR gain. Additionally, blockade of spontaneous PF-PC LTD was completely compensated by overinduction of train-induced LTD. Notably, the more increment in this case does not mean the greater final OKR gain than normal, since the initial state of OKR gain started from 0 rather than 0.3. **(Middle)** Bar graph showing the short-term or fast adaptation of OKR gain measured by gain differences before and after 1 h of training on the 5th day. Dashed lines indicate normal values. **(Bottom)** Bar graph showing the percentage of synaptic weight changes at the fifth session of learning.

Complete interruption of the information flow from the cerebellar input layer to the output layer cannot address the reason of the enormous amount of GCs. Therefore, to better understand the evolutionary conservation of this abundant number of cerebellar GCs, a more desirable strategy would be to suppress the neurotransmission from most, but not all, GCs. Moreover, since an experimental elimination of GCs can cause morphological processes in their target neurons (Galliano et al., 2013), it is likely profitable to investigate the functional outcome of inactivating the majority of GCs computationally rather than experimentally. In the first lesion scenario, we have attenuated transmission at MF-GC synapses by reducing α from 1 to 0.3. Based on the model formulation (Equations 9, 10, 15, and 16), the impact of α reduction is not limited to the input layer but propagated in the plasticity rules of the next layers. As a consequence of this manipulation, the overall gain of OKR and plasticity of the synapses significantly declined (Figure 3). To provide a tighter comparison of normal and lesion scenarios, we have depicted short- and long-term gain increment of the fifth session of training besides the percentage of synaptic weight changes of the same day in Figure 6. Positive and negative long-term gain increments are respectively, considered as the occurrence of motor learning and disruption of basic motor performance. Considering Figure 6, it can be seen that even 70% attenuation in MF-GC transmission did not disrupt basic motor performance although motor learning and memory consolidation were compromised. This result is in line with a previous study from Galliano et al. (2013) who noted a minority of functionally intact GCs is adequate for the maintenance of basic motor performance, whereas formation and consolidation of sophisticated memories require higher numbers of normal GCs regulating PC firing.

LTD at PF-PC synapses is believed to be the main cellular mechanism for cerebellar motor learning (Kano et al., 1994; Ito, 2001; Belmeguenai et al., 2008; Hirano, 2013; Yuzaki, 2013; Kuwabara et al., 2014; Freeman, 2015). However, the necessity of LTD for the acquisition of motor learning is challenged by the demonstration of normal motor learning in LTD-deficient mice (Welsh et al., 2005; Faulstich et al., 2006; Schonewille et al., 2011). In response to the dichotomy that has arisen between PF-PC LTD and motor learning, some researchers proposed LTP as an alternative substrate for motor learning (Schonewille et al., 2010), some others have spoken about the possibility of a compensatory pathway (Lemon and Edgley, 2010; Gao et al., 2012), and some found the existing documentation inadequate and postponed the final conclusion to further *in vivo* experiment and theoretical investigations (Hirano, 2013; Ito et al., 2014). In this regard, we conducted a multi-stage lesion scenario to examine the possible involvement of different factors (including train-induced LTD, spontaneous LTD, spontaneous LTP, and rate of learning and recovery) in weight changes at PF-PC synapses and motor learning. The impairment of train-induced PF-PC LTD is simulated by partial and complete abrogation of temporal correlation between use-dependent and synchronous activity of CF and PFs (see details in Table 1). Figure 4B.1 compares the OKR gain and synaptic weights in the partial and complete lesion conditions during five sessions of learning. The OKR gain continued to increase at a lower rate than normal in the partial lesion case, whereas it remained largely stopped at the initial state in the complete lesion case. This result indicates that complete blockade of train-induced PF-PC LTD would interrupt motor learning without causing noticeable damage to baseline motor performance. Furthermore, deficiency in train-induced PF-PC LTD led to a 31% increase in PF-MLI synaptic weight in the complete lesion case, which might be interpreted as a confirmation for the compensatory role of PF-MLI pathway for PF-PC synapse. However, despite the major impact of such a compensatory mechanism in upregulating the MF-DN synaptic weight (*W*_{*MF*−*DN*}_) and hindering fall from the initial gain state (0.3), it was not that enough to prevent loss of motor learning. Therefore, PF-MLI synapses contribute in providing a more robust basic motor performance rather than compensating motor learning. As shown in Figure 6, the blockade of train-induced LTD had a negative impact on both short- and long-term gain increments. Specifically, in the complete lesion case, the slight increase in the short-term gain results from the modulating effect of MLIs on PCs. Therefore, it can be inferred that the molecular layer takes part in short-term memory formation, but not as much as Purkinje layer does. Next, we simulated spontaneous PF-PC LTD deficit by eliminating LTD induced by spontaneous activity of CF and PFs. As a result, the baseline weight of *W*_{*PF*−*PC*}_ became larger than normal. The increase in *W*_{*PF*−*PC*}_ overactivates PCs and takes *W*_{*MF*−*DN*}_(*t*) to 0. As shown in Figure 4B.2, the initially decreased OKR gain was successfully compensated day by day through overinduction of train-induced LTD. According to Figure 6, one of the highest rate of train-induced PF-PC LTD induction with 44% depression in *W*_{*PF*−*PC*}_ and the highest rate of short- and long-term gain increment among all the lesion scenarios belong to blockade of spontaneous LTD. This shows the high ability of train-induced PF-PC LTD in compensating spontaneous PF-PC LTD deficit. It seems that the behavioral learning differences between spontaneous and train-induced LTD are the key to explain the discrepancies between cerebellar PF-PC LTD and motor learning. A conventional protocol of PF-PC LTD induction is composed of conjunctive stimulation of PFs and CF at 1 Hz for 5 min or pairing PFs stimulation with direct intracellular depolarization of PC (Hammond et al., 2015); this stimulation corresponds to spontaneous LTD, since both CF and PFs elicit tetanic spikes without temporal modulation, while train-induced LTD is provoked by use-dependent and variable activity of CF and PFs (Schonewille et al., 2011). Then, we simulate spontaneous PF-PC LTP by inactivating LTP induction at PF-PC synapses, which was triggered via the background activity of GCs. This manipulation disturbed the balance of *W*_{*PF*−*PC*}_ baseline in favor of spontaneous LTD and decreased *W*_{*PF*−*PC*}_ so that it was clamped to 0 and consequently train-induced LTD was also inactivated. In this condition, the OKR gain did not increase after five sessions of learning (Figure 4B.2). The long-term gain increment has a slight (non-significant) negative value (Figure 6), which indicates the intactness of basic motor performance despite the complete destruction of motor learning and memory consolidation. This result is in line with the experimental observations in calcineurin-deficient mice (Schonewille et al., 2010). The slight formation of STM, in this case, is related to MLI activity.

Next, we carried out the simulation with two different learning and recovery rates for the synaptic weight at PF-PC synapses to specify the impact of early and late phases of LTD on short- and long-term memory and to check the robustness of suggested plasticity rule against parameter variation. As shown in Figure 6, increasing the recovery rate of *W*_{*PF*−*PC*}_ reduced long-term gain increment although there was no defect in short-term gain increment. It seems that shortening the duration of late phase of LTD (increasing recovery rate) had a negative impact on transfer and subsequent consolidation of motor learning. On the other hand, decreasing the learning rate of *W*_{*PF*−*PC*}_ (lengthening the duration of the early phase of LTD) had a negative impact on short-term memory formation, which is a prerequisite for long-term consolidation. Furthermore, application of different sets of learning or recovery rate did not cause any unrealistic performance overshoot or disruption in the order of LTD/LTP occurrence in *W*_{*PF*−*PC*}_ and *W*_{*MF*−*DN*}_, which shows the robustness of the suggested plasticity rule against parameter variations.

In the third lesion scenario, we considered partial and complete blockade of CF signal that emanated from IO neurons to the cerebellar cortex by inactivating train-induced plasticity at both PF-PC and PF-MLI synapses. The model ability in reproducing lesioned behavior was successfully verified by experimental data (Katoh et al., 1998; Shutoh et al., 2006) [compare the simulated OKR gain of complete IO lesion with the experiment (Figure 4C)]. After lesions were made on the olivary system, the ability to change OKR gain was significantly reduced compared with the normal group. In the complete block case, both short- and long-term gain increment had negative values (Figure 6). This suggests that the olivary system (specifically CFs) plays a critical role not only in motor learning but also in basic motor performance. As a confirmation for this view, mutant mice with structural abnormalities in the innervation pattern of PCs by CFs have shown obvious signs of ataxia (Janahmadi et al., 2009; Yuzaki, 2013). Compared with the train-induced PF-PC lesion, complete blockade of CF had a more negative impact on basic motor performance. A possible factor that may have led to such a difference is the lack of train-induced PF-MLI LTP in the CF lesion case. This issue supports this notion that train-induced PF-MLI LTP plays a crucial role in providing a more robust basic motor performance.

Although feedforward inhibition of MLIs onto PCs have been studied extensively, its behavioral relevance has remained enigmatic (Wulff et al., 2009). In the fourth lesion scenario, we investigated how GABAergic neurotransmission from MLIs to PCs regulates adaptation of OKR by conducting the simulation in two modes: (1) no PF-MLI plasticity and (2) no PF-MLI baseline (Figure 4D). In the no plasticity mode, we assumed that PF-MLI synaptic weight is fixed at a baseline value ($W_{\left\{PF-MLI\right\}}^{0}$). The resulting OKR gain did not show any considerable difference with that of the normal group. The lack of plasticity in PF-MLI synapse was successfully compensated by about 5% overinduction in train-induced PF-PC LTD. The question that may be posed is that if the lack of plasticity in the PF-MLI synapse does not cause any problems in the learning process, what then is the role of this plasticity? The plasticity that was induced at PF-MLI synapses relies on indirect (spillover) activity of CFs (Gao et al., 2012). Thus, it may have a lower learning capacity and less effective power to modulate the activity of DN and the final output of the cerebellum. However, as predicted earlier, the little memory that forms in this plasticity is used to provide a robust basic motor performance especially when train-induced PF-PC LTD is absent. In the no baseline mode, we set the baseline of *W*_{*PF*−*MLI*}_ at 0. Removing the inhibition from PCs increased their excitability and shifted the membrane potential of DN toward a hyperpolarized state. To avoid the overexcitation of PCs and unrealistic variations in PF-MLI plasticity, we decreased the baseline value of *W*_{*PF*−*PC*}_ and transmission coefficient of *W*_{*PF*−*MLI*}_, respectively. Simulation results showed that the daily increase in OKR gain did not accumulate by the repetition of training sessions (Figure 4D). This suggests that blockade of PF-MLI baseline activity impairs memory transfer and consolidation, leaving short-term memory formation intact, consistent with experimental reports (Wulff et al., 2009).

In the last lesion scenario, we blocked PC-DN transmission by setting γ = 0 immediately after training on the fourth session of learning. An experimental data set (Shutoh et al., 2006) is used to test the ability of the model in reproducing the lesion behavior (Figure 5). This manipulation caused almost all that has been learned in the fourth session to be lost and gained back to its previous level. Furthermore, deactivation of the PC-DN pathway eliminates the induction of LTP at the MF-DN synapse, indicating that memory transfer from *W*_{*PF*−*PC*}_ to *W*_{*MF*−*DN*}_ is disrupted. Normally, the disruption of memory transfer affects long-term learning consolidation. This suggests that the cerebellar cortex plays a critical role not only in short-term memory formation but also in transferring the formed memory to the nuclear region. Furthermore, it can be inferred that OKR gain depends on the modulation strength of PC activity. In an extreme case when all PF–DN synapses die out, PCs cannot modulate their spike discharges, thereby decreasing the OKR gain.

## Discussion

In this study, we presented a mechanistic firing rate model of the cerebellar circuit endowed with bidirectional plasticity rules located at PF-PC and PF-MLI synapses and a Hebbian-like plasticity rule with a postsynaptic gating mechanism at MF-DN synapses. Considering the synaptic interaction among the cerebellar layers, the concept of transmission coefficient was proposed as a tool for motor learning analysis. Manipulation of transmission coefficients at five synaptic junctions among cerebellar layers, referred to as lesion scenarios, provided a great potential to answer some of the fundamental questions about the underlying mechanisms involved in cerebellar motor learning and memory formation.

In order to select a modeling strategy in compliance with the objectives of this research, the following points were considered. (*i*) We tried to adhere to the structure and physiology of the cerebellum and, at the same time, preserve the simplicity of the model. This simplicity helped us to provide a panoramic view of the different phases of motor memory formation and to reproduce steady-state changes of learning behavior over a day time scale. (*ii*) The adaptation of OKR gain as a cerebellar-dependent task is used to make a more straightforward relationship between the neural and behavioral domains. This improves the interpretability of behavioral disorders caused by cerebellar malfunction and minimizes the possible involvement of other brain regions in the control of movement. (*iii*) Synaptic plasticity rules were defined as a function of background and use-dependent activities of the neuronal population. Distinguishing between the underlying factors involved in long-term synaptic weight modification helped us to further elucidate their contribution to the learning process. (*iv*) The compatibility of the simulation results with experiments was examined. The results showed good accordance with the findings of previous studies. (*v*) The functional contribution of interneurons in motor learning was regarded. (*vi*) The possibility of a compensatory mechanism in different lesion scenarios was investigated.

The repetitive alternation between task and off-task period during gain-up training for five consecutive sessions effectively increased the OKR gain by 90%. Meanwhile, the model could show the cooperative action of the main forms of synaptic plasticity at PF-PC, PF-MLI, and MF-DN synapses during learning. Then, we tested the model performance under various lesion conditions. The percentage of changes in synaptic weights and the extent of gain increment relative to the initial state were used as a measure for evaluating and comparing different conditions. The model predicted that train-induced PF-PC LTD is the most effective compensation mechanism since it could successfully cover the lack of spontaneous PF-PC LTD and PF-MLI plasticity. In addition, train-induced PF-PC LTD had a major role in the learning capability of the model since its blockade completely stopped motor learning and memory formation, although it did not disrupt basic motor performance. In this regard, the overinduction of PF-MLI LTP was not able to prevent motor learning impairment, despite its vital role in preserving the robustness of basic motor performance. The pivotal role of PF-PC LTD in cerebellar learning is highlighted in several studies, including those evaluating the animal models of autistic spectral disorder (ASD) (Koekkoek et al., 2005; Baudouin et al., 2012; Piochon et al., 2014). For instance, the incomplete elimination of surplus CFs in patDp/+ mice causes PF-PC LTD saturation and motor learning impairment (Piochon et al., 2014). Similarly, deletion of Fmr1 gene in PCs (L7-Fmr1; a model for syndromic autism) attenuates eyeblink conditioning by altering LTD profile (Koekkoek et al., 2005). ASD models with environmental etiology also support the involvement of synaptic transmission of PCs in motor learning (Wang et al., 2018). Valproic acid (VPA) is an anti-seizure drug that can replicate ASD-like symptoms. Administration of VPA effectively reduces the cell density and dendritic arborization of PCs. Such an anatomical defect is associated with suppressed synaptic transmission of PCs and impaired motor learning in VPA-treated mice (Wang et al., 2018). As another lesion case, blockade of spontaneous PF-PC LTP clamped *W*_{*PF*−*PC*}_ to 0 and subsequently disabled motor learning. Of course, as in the previous case, this manipulation did not show abnormal motor performance. Thus, it can be inferred that both train-induced LTD and spontaneous LTP are required for motor learning. If the motor performance is considered as a consequence of postnatal long-term learning (Manto and Jissendi, 2012), then the birth defect in train-induced LTD or spontaneous LTP would cause severe impairment in motor performance. Compared to LTD, fewer studies have focused on the impact of PF-LTP on motor learning (Yuzaki, 2013). L7-PP2B mutant mouse is one of the animal models created for this purpose (Schonewille et al., 2010). Consistent with our results, this mutant showed impaired acquisition of eyeblink conditioning and impaired gain adaptation of vestibulo-ocular–reflex. The basal motor performance remained unaffected. Another point about the mutant is that, despite the abrogation of LTP, LTD functions normally. This may appear to be in conflict with our results and the notion of mutual dependence between LTD and LTP suggested by several studies (Baudouin et al., 2012; Yuzaki, 2013). However, our model explained this contradiction by differentiating between train-induced and spontaneous LTD and predicted that the reported LTD, induced by frequency constant stimulus, was spontaneous and not train-induced. Among all the lesion cases, IO lesion, which was referred to as blockade of CF signaling to cerebellar cortex, had the most destructive effect on both motor learning and basic motor performance since it would result in the inactivation of all train-induced mechanisms mediated by CF error signal not just LTD in PF-PC, but also LTP in PF-MLI synapses. Thus, studies on transgenic animals that suffer from IO lesion or CFs abnormalities may not be an exact representation of LTD defect. As another case, the lesion caused by 70% attenuation of MF-GC transmission did not disrupt basic motor performance although it compromised motor learning and memory consolidation. Based on our formulation, manipulating the transmission coefficient in the input layer reduced the plasticity of synaptic weights in the next layers and even the inner nuclear layer. These observations provide important clues from a dynamical viewpoint, as to why vertebrates require an extreme abundance of functional GCs in their life and to what extent this abundance is redundant. The functionality of GABAergic interneurons in regulating the adaptation of OKR was investigated in two different conditions: (1) no PF-MLI plasticity and (2) no PF-MLI baseline. The model predicted that the lack of PF-MLI plasticity is completely compensable through overinduction of train-induced PF-PC LTD. Furthermore, the indirect access of MLIs to instruction signals from CFs and the final output of the cerebellum reduce their learning and modulating capability. The necessity of PF-MLI plasticity was more tangible when the train-induced PF-PC LTD had defected. In such a circumstance, PF-MLI plasticity acted as a reserve mechanism, using its limited short-term memory formation capacity to save the basic motor performance from impairment. On the other hand, lack of PF-MLI baseline impaired memory transfer and consolidation, while leaving short-term memory formation intact. Simulation of the learning process with different sets of learning and recovery rates asserted that the early phase of LTD participates in short-term memory formation and the late phase of LTD is involved in memory transfer. The possibility of a hierarchical causality in transferring the acquired short-term memory across the cortical and its partial consolidation into the nuclear parts of the cerebellum was investigated by cutting off the connection of flocculus with the DN during the training. After lesions were made, OKR gain returned to its pretraining level and MF–DN synaptic weight failed to further increase, whereas PF–PC synaptic weight remained intact, indicating disruption of memory transfer and consequently long-term memory consolidation. The existence of such a biphasic mechanism, with a first rapid phase of memory formation in the cerebellar cortex and a slower phase of memory consolidation in the cerebellar nuclei, is also observed in the spiking models of the cerebellum (Antonietti et al., 2016, 2017; Luque et al., 2016). These models showed that the functional interplay among distributed plasticity sites could facilitate the slow memory consolidation at the nuclear sites, which is important for faster and more stable reacquisition of associative motor tasks. Recently, a distributed spiking model of the cerebellum is used to predict the impact of different pathological damages of the cerebellar cortex on the acquisition of eyeblink conditioning (Geminiani et al., 2018). The pathological cases consistently indicated that (1) an intact functionality of cerebellar cortex is needed to accelerate memory transfer to DN; (2) partial impairment in MF afferents results in an imperfect transmission of information to the granular layer, which in turn influences the activity of PCs and compromises learning; and (3) partial damage in PF-PC LTD does not completely stop learning, but decreases its velocity.

**Figure 7:**
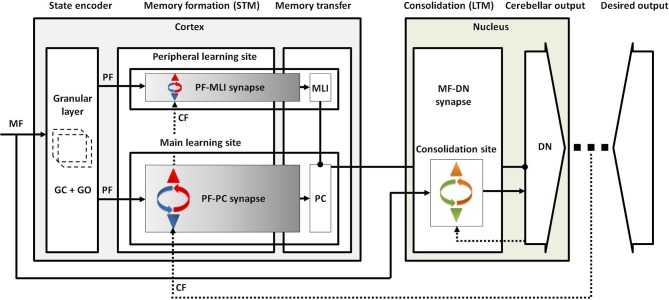
Conceptual map of procedural motor memory formation in the cerebellum. Sensory input is transmitted to the cortical and nuclear modules by MFs. The granular layer, benefiting from abundant clusters of granule and Golgi cells, conveys the encoded sensory information to both molecular and Purkinje layers by PFs. Conjunctive activation of PFs and CF forms short-term memory at the main and peripheral learning sites. Then, the formed memory is transferred to the nuclear module through inhibition exerted by PCs on DN mostly at the late phase of LTD. Finally, the postsynaptic activity of DN drives the formation of long-term memory at the consolidation site.

Taking all the obtained results from lesion scenarios together, we proposed a conceptual map for procedural motor memory formation in the cerebellum (Figure 7). Accordingly, the sequential processes of cerebellar motor learning occur in the following way. (1) Conjunctive activation of PFs and CF during training sessions causes short-term memory formation at PF-PC synapses (main learning site) and to a lesser extent at PF-MLI synapses (peripheral learning sites). (2) After training, the activity of DN is modulated to a higher level than before training, since the inhibition exerted by PCs is weakened by PF-PC LTD and PF-MLI LTP. (3) This enhanced DN activity resulted in LTP at MF-DN synapses by a Hebbian-like postsynaptic mechanism. (4) Meanwhile, PF-PC synapses (along with PF-MLI synapses) recover from LTD steadily by spontaneous PF-PC LTP, and this causes a slow decrease in DN activity. Thus, the late phase of LTD provides enough time to transfer memory from PF-PC to MF-DN synapse. (5) The recovery from PF-PC LTD and PF-MLI LTP erases the learned short-term memory, whereas (6) the slow decrease of DN activity acts to consolidate learning at MF-DN synapses. When the train-induced PF-PC LTD defects, PF-MLI plasticity acts as a backup mechanism, using its limited short-term memory formation capacity to save the basic motor performance from impairment.

It should be noted that, although long-term synaptic plasticity has been proposed to be the dominant form of plasticity for cerebellar motor learning, various types of plastic mechanisms can occur at multiple synaptic or even non-synaptic sites of the network that provide supplementary and overlapping functions (Gao et al., 2012). For instance, there is accumulating evidence supporting the involvement of short-term and intrinsic plasticity in motor learning and memory formation (Hirano, 2018; Shim et al., 2018). However, the exact functionality of such phenomena remains to be shown. Furthermore, the transient nature of the short-term plasticity and the non-synaptic (non-local) character of the intrinsic plasticity make the tracking of their effects harder. Therefore, analyzing the steady-state behavior of the system, we ignore the short-term plasticity and mainly focus on the effect of long-term synaptic changes on cerebellar learning. We also implicitly take into account the role of the intrinsic plasticity in determining the optimum working frequency and basal intrinsic excitability of PCs by modifying the corresponding baseline values of synaptic weights in the lesion conditions.

The selected modeling strategy has a great ability in displaying the equilibrium behavior of the system, even in the post training period. However, this simplified model, working with analog variables representing the average firing rates of the different neuronal populations, encounters limitations in showing the spatiotemporal evolution of spike patterns of the principal neurons and the diversity of delays imposed by the interneurons. Equipping the model with the spike-timing-dependent plasticity (STDP) mechanisms allows us to match learning with the network temporal dynamic and to simulate more complex phase-varying tasks.

In future work, the impact of different learning paradigms on the efficacy of learning can be evaluated. Also, to gain further insight into the cellular and molecular bases of motor learning, it is necessary to extend the compartments of the model to mimic the non-linear behavior of cerebellar network more precisely. In this study, we considered gain adaptation of OKR as a representative of cerebellar learning. Certainly, the study of model behavior in the implementation of other cerebellar-dependent tasks can help to uncover more features of motor learning in the cerebellum.

Altogether, the proposed model, taking the efficacy of neurotransmission among different layers of the cerebellum into account implicitly, provides a computational basis toward evaluating multiple synaptic plasticity in cerebellar learning and memory.

## Data Availability

The authors confirm that the data supporting the findings of this study are available within the article.

## Author Contributions

SS, FB, and MJ conceived the work. SS developed the codes and performed the computations. SS, FB, and MJ analyzed and interpreted the results. SS wrote the manuscript in consultation with FB and MJ.

### Conflict of Interest Statement

The authors declare that the research was conducted in the absence of any commercial or financial relationships that could be construed as a potential conflict of interest.
